# Thymic stromal lymphopoietin gene promoter polymorphisms and expression levels in Graves’ disease and Graves’ ophthalmopathy

**DOI:** 10.1186/1471-2350-13-116

**Published:** 2012-11-30

**Authors:** Kun-Hsi Tsai, Fuu-Jen Tsai, Hui-Ju Lin, Hung-Jung Lin, Yu-Huei Liu, Wen-Ling Liao, Lei Wan

**Affiliations:** 1 Department of Emergency Medicine, Chi Mei Hospital, Liouying, Tainan, Taiwan; 2 Genetic Center, China Medical University Hospital, 404, Taichung, Taiwan; 3 School of Post Baccalaureate Chinese Medicine, China Medical University, Taichung, Taiwan; 4 Department of ophthalmology, China Medical University Hospital, Taichung, Taiwan; 5 Graduate Institute of Integrated medicine, China Medical University, Taichung, Taiwan; 6 School of Chinese Medicine, China Medical University, Taichung, Taiwan; 7 Department of Biotechnology, Asia University, Taichung, Taiwan

**Keywords:** Graves’ disease, Graves’ ophthalmopathy, Thymic stromal lymphopoietin, Th17

## Abstract

**Background:**

Graves disease (GD) is an organ-specific autoimmune disease characterized by hyperthyroidism, diffuse goiter, autoantibodies against thyroid-specific antigens, and dermopathy. Studies of GD have demonstrated the importance of the Th2 and Th17 immune responses in mediating disease progression. In the present study, we investigated the role of a Th2 cytokine, thymic stromal lymphopoietin (TSLP), in GD and Th17 differentiation.

**Methods:**

In this study, we genotyped 470 patients with GD at 3 single nucleotide polymorphisms (SNPs) in *TSLP*. In addition, the serum concentrations of TSLP were determined in 432 patients and 272 controls. Ten patients and controls each were further screened using in vitro Th17 differentiation assays. The SNPs were genotyped using ABI TaqMan® SNP genotyping assays. For the Th17 differentiation assays, peripheral blood mononuclear cells (PBMCs) isolated from the patients and controls were placed into Th17 differentiation media, and interleukin 17 expression levels were determined.

**Results:**

Haplotype analysis indicated that patients with the Ht3 (TCC) haplotype have a 3.28-fold higher risk of developing GD (*p* = 0.007), whereas those with the Ht5 (TCG) haplotype had a 0.03-fold, reduced risk of developing GD (*p* = 1 × 10^−14^). SNP rs3806933 (*p* = 0.007) was associated with female Graves ophthalmopathy (GO). TSLP expression levels were higher in GD patients than in control subjects, and TLSP was also shown to promote the differentiation of Th17 cells in GD patients.

**Conclusions:**

These results suggest that polymorphisms in *TSLP* may be used as genetic markers for the diagnosis and prognosis of GD. Furthermore, TLSP may be a target for treating GD.

## Background

Graves’ disease (GD) is a complex, organ-specific autoimmune disease characterized by a variety of clinical features, such as hyperthyroidism, diffuse goiter, the presence of autoantibodies against thyroid-specific antigens, and dermopathy. The dysregulation of Th1 and Th2 responses in patients with GD are associated with progression of the disease. GD patients often present with anti-thyrotropin receptor antibodies (TRAb)—an immune response regulated by Th2 cells—which stimulate a variety of biological responses that lead to hyperthyroidism and goiter [1]. Moreover, the recurrence of GD after treatment with antithyroid drugs increases serum immunoglobulin E and interleukin 13 (IL-13) levels, which are typical features of a Th2 immune response [1].

Thymic stromal lymphopoietin (TSLP) is a member of the hematopoietic cytokine family and is produced by epithelial cells, keratinocytes, and granulocytes. Several cellular targets of TSLP have been identified, including dendritic cells, lymphocytes, and granulocytes. TSLP-stimulated dendritic cells are able to activate CD4^+^ T cells in an antigen-specific manner, resulting in T cells with the Th2 phenotype that produce proallergic cytokines (IL-4, IL-5, IL-13, and TNF-α), while down-regulating IL-10 and IFN-γ [2]. Studies of TSLP in humans have indicated its potential role in the Th2 inflammatory response. TSLP is an important factor in the pathogenesis of asthma and a potential therapeutic target for the treatment of allergic diseases [3,4]. Recent human studies have shown that IL-17 is expressed in the airways of patients with asthma and that IL-17 expression is greater in patients with moderate to severe asthma compared with patients with mild asthma and healthy subjects. TSLP and toll-like receptor (TLR) 3 ligands activate CD11^+^ dendritic cells in human blood to produce IL-23 and program naive CD4^+^ T cells to differentiate into Th17 cells. TSLP-mediated Th17 cell commitment under Th2-polarizing conditions may be involved in the development of an inflammatory mixture of Th2 and Th17 cells, a unique profile that resembles severe asthma [5]. The proportion of Th17 cells in GD patients is higher than that in control subjects [6,7]. Th17 cells thus play an important role in GD and other inflammatory autoimmune disorders, including multiple sclerosis [8,9], rheumatoid arthritis [10,11], and Crohn’s disease [12]. In rheumatoid arthritis (RA) patients, the expression levels of TSLP and IL-17 are increased in the synovial fluid. TSLP was shown to promote Th17 differentiation and enhance arthritis in murine rheumatoid arthritis models, indicating the importance of TSLP in the differentiation of Th17 and the involvement of TSLP in the pathogenesis of autoimmune disease [13].

The role of TSLP in Th17 cell differentiation implicates TSLP in the pathogenesis of GD. However, TSLP has not been directly investigated in the context of GD; its role in the disorder thus remains unclear. In the present study, we indicated that TSLP polymorphisms are associated with GD and that expression levels of TSLP are higher in patients than in control subjects. In addition, we showed that TSLP mediates the differentiation of CD4^+^ T cells into Th17 cells.

## Methods

### Patients

A total of 470 patients with GD were enrolled in this study. The diagnosis of GD was performed by endocrinologists and Graves’ ophthalmopathy (GO) was assessed by ophthalmologists. The diagnosis of GD was made on the basis of clinical symptoms and biochemical affirmation of hyperthyroidism, multinodular goiter, and a positive result for at least one of the following biochemical tests: thyroid-stimulating hormone receptor antibody, diffusely increased iodine-131 uptake in the thyroid gland, and exophthalmos. Patients with GD were classified in accordance with the NOSPECS system recommended by the American Thyroid Association. In GD patients, GO was identified by proptosis, with or without a more severe phenotype (classes 3–6). Proptosis was measured by a Hertel exophthalmometer and was defined as the anteroposterior protrusion of the globe by more than 19 mm from the lateral orbital rim in either eye, or by any discrepancy in the degree of protrusion of the 2 eyes of more than 1 mm. The demographic and clinical characteristics of control subjects and Graves’ disease patients are listed in Additional file 1: Table S1. We selected gender- and age-matched controls for the association study, the functional assay and the in vitro IL-17 differentiation experiments. The patient and control groups of in vitro IL-17 differentiation experiment each comprised 2 male and 8 female individuals, and both groups had mean ages of 38.42 ± 10.91 and 40.12 ± 8.48 years, respectively. Blood samples were collected for genomic DNA isolation and serological tests. This study was approved by the ethical committee and institutional review board of the China Medical University Hospital. Informed consent was obtained from all patients or their guardians.

### SNP genotyping and ELISA

TSLP SNP genotype information was retrieved from the HapMap database for the HCB + JPT populations. Tagging SNPs were selected through the Tagger function in the Haploview software program. The selection criteria were: (1) the minor allele frequency should be greater than 10%; (2) SNPs that potentially affect transcription efficiency, translation efficacy, or protein functions were selected first; and (3) the availability of probes or primers that pass the manufacturer’s qualification requirements (Applied Biosystems Inc., Foster City, CA). Only the following 4 SNPs fulfilled the criteria: rs3806933, rs2289276, rs2289278, and rs11466741. The rs3806933 and rs2289276 SNPs are located in the promoter region and rs2289278 is located in intron 2. Genotyping rs3806933, rs2289276, and rs2289278 captured 4 of 4 (100%) alleles with r^2^ ≥ 0.8. SNPs were genotyped using ABI TaqMan® SNP genotyping assays (rs3806933: C_3166722_10; rs2289276: C_3166723_20; rs2289278: C_15880989_10; Applied Biosystems) on a Roche LightCycler®480 system (Roche). Haplotypes were inferred from unphased genotype data using the Bayesian statistical method in the Phase 2.1 software program. All 3 SNPs were analyzed using Phase 2.1 software. TSLP concentrations in sera were determined with ELISA kits (eBioscience Inc.).

### Th17 differentiation assay

Peripheral blood mononuclear cells (PBMC) were isolated from 10 healthy individuals (5 male and 5 female subjects) and 10 Graves’ disease patients (5 male and 5 female patients), and cultured in RPMI1640 medium containing 10% fetal bovine serum. Stimulation was performed with plate-bound anti-CD3 and anti-CD28 (1 μg/mL and 1 μg/mL, respectively). A total of 1.2 × 10^4^ PBMC were plated into each well of a round-bottom 96-well plate. The media contained 10 ng/mL of TGF-β, 10 ng/mL of IL-23, 10 ng/mL of IL-6, 10 ng/mL of IL-1β, 1 μg/mL of anti-IL4 and anti-IFNγ, with or without 125 ng/mL of TSLP, and incubated at 37°C with 5% CO_2_ for 5 days. IL-17 concentrations were determined by ELISA.

### Statistical analysis

IBM SPSS statistics software version 20.0 (IBM Corporation) was used to analyze the data. The genotype frequency and allelic frequency distributions of the polymorphisms in patients with GD (with or without GO) were analyzed by the chi-square method. The difference in sera TSLP concentrations between control subjects and patients was analyzed by *t*-test. A p-value less than 0.05 was considered statistically significant. The odds ratio (OR) was calculated from genotype frequencies and allelic frequencies with a 95% confidence interval (CI).

## Results

To investigate potential associations between TSLP and GD, 3 polymorphisms in the *TSLP* gene were selected for this study. A total of 470 patients and 78 control subjects were genotyped at these SNPs. Genotype frequencies from our GD cohort were then compared with those of the normal healthy population. The rs2289276 SNP was found to be associated with GD when compared with the general population (recessive model, *p* = 0.027; Table 1). Statistical significance was lost when adjusted for multiple comparisons (Bonferroni correction; *p* < 0.016). Haplotype analysis indicated that patients with the Ht3 (TCC) haplotype have a 3.28-fold higher risk of developing GD (*p* = 0.007), whereas those with the Ht5 (TCG) haplotype had a 0.03-fold reduced risk of developing GD (*p* = 1 × 10^−14^) (Table 2). We then grouped the GD patients on the basis of the presence or absence of GO to determine whether *TSLP* polymorphisms were associated with the GO phenotype. The rs3806933 SNP was found to be associated with GO under a genotypic model (*p* = 0.028). Using a dominant model comparison, rs3806933 (*p* = 0.021; OR, 1.54; 95% CI: 1.07, 2.23) and rs2289278 (*p* = 0.045; OR, 1.47; 95% CI: 1.01, 2.14) were both associated with GO when compared with patients without GO. In addition, rs2289276 was associated with GO under a recessive model (*p* = 0.026; OR, 2.98; 95% CI: 1.09, 8.12; Table 1). Statistical significance was lost when adjusted for multiple comparisons (Bonferroni correction; *p* < 0.016). Both rs2289276 and rs3806933, which are located in the promoter region of *TSLP*, are known to influence *TSLP* expression [14]. However, no significant differences in allele frequencies were observed between patients with GO and those without GO or between patients with GO and the normal population (Table 1).

**Table 1:** Genotype and allele frequencies of markers for Graves’ disease patients in Taiwan

**SNP ID**	**GD with GO (201)**	**GD without GO (269)**	**GD (470)**	**Control (78)**	***P***^a^	***P***^b^	**OR (95% CI)**
	**N (%)**	**N (%)**	**N (%)**	**N (%)**			
**Genotype**							
**rs3806933**							
C/C	83(41.3)	140 (52.0)	223 (47.4)	37 (47.4)			1
C/T	101 (50.2)	102 (37.9)	203 (43.2)	31 (39.7)			1.67 (1.13, 2.46)
T/T	17 (8.5)	27 (10.0)	44 (9.4)	10 (12.9)	0.028 (genotype)	0.61	1.06 (0.55, 2.06)
C/T + T/T	118 (58.7)	129 (48.0)	247 (52.6)	41 (52.6)	0.021 (dominant)	1	1.54 (1.07, 2.23)
**rs2289276**							
C/C	5 (2.5)	19 (7.1)	24 (5.1)	9 (11.5)			1
C/T	77 (38.3)	90 (33.5)	167 (35.5)	29 (37.2)			3.25 (1.16, 9.12)
T/T	119 (59.2)	160 (59.5)	279 (59.4)	40 (51.3)	0.065 (genotype)	0.067	2.83 (1.03, 7.79)
C/T + C/C	196 (97.5)	250 (92.9)	446 (94.9)	69 (88.5)	0.026 (recessive)	0.027	2.98 (1.09, 8.12)
**rs2289278**							
C/C	114 (56.7)	177 (65.8)	291 (61.9)	41 (52.6)			1
C/G	73 (36.3)	79 (29.4)	152 (32.3)	32 (41.0)			1.43 (0.97, 2.13)
G/G	14 (14)	13 (4.8)	27 (5.8)	5 (6.4)	0.125 (genotype)	0.29	1.67 (0.76, 3.69)
C/G + G/G	87 (50.3)	92 (34.2)	179 (38.1)	37 (47.4)	0.045 (dominant)	0.12	1.47 (1.01, 2.14)
**Allele**							
**rs3806933**							
C allele	267 (66.4)	382(71.0)	649 (69)	105 (67.3)			1
T allele	135 (33.6)	156 (29.0)	291 (31)	51 (32.7)	0.13	0.66	1.24 (0.94, 1.64)
**rs2289276**							
T allele	315 (78.4)	410 (76.2)	725 (77.1)	109 (69.9)			1
C allele	87 (21.6)	128 (23.8)	215 (22.9)	47 (30.1)	0.44	0.05	0.88 (0.65, 1.21)
**rs2289278**							
C allele	301 (74.9)	433 (80.5)	734 (78.1)	114 (73.1)			1
G allele	101 (25.1)	105 (19.5)	206 (21.9)	42 (26.9)	0.04	0.17	1.38 (1.01, 1.89)

Abbreviations: CI, confidence interval; GO, Graves’ ophthalmopathy; SNP, single-nucleotide polymorphism. ^a^Differences in allele, dominant or recessive genotype frequencies were determined by chi-square test using 2 × 2 contingency tables. Differences in genotype frequencies were determined by chi-square test using 2 × 3 contingency tables. Compared Graves’ diseases patients with and without GO. ^b^ Compared Graves’ disease patients with normal population. *P* values less than 0.016 were considered significant.

**Table 2:** The association between TSLP gene haplotypes among control and GD patients

Haplotype*	**rs3806933**	**rs2289276**	**rs2289278**	**Control**	**GD**	*p* value**	**OR (95% CI)**
Ht1	C	C	C	60	433	0.09	1.35 (0.95–1.9)
Ht2	C	C	G	37	205	0.56	0.89 (0.59–1.32)
Ht3	T	C	C	5	93	0.007	3.28 (1.31–8.21)
Ht4	T	T	C	39	200	0.27	0.8 (0.54–1.19)
Ht5	T	C	G	12	2	1 × 10^−14^	0.03 (0.01–0.11)

* The haplotypes were identified by the Bayesian statistical method available in the program Phase 2.1.

** The chi-square test (2 × 2 table) was performed to obtain the *p* value. For example, the numbers of haplotypes of control and GD with Ht1 were compared with the numbers of control and GD without Ht1. A *p* value < 0.01 was considered as statistically significant. Only haplotype with a frequency of 0.01 or above in either cases or controls are shown.

In this study, there were 374 female and 96 male patients. Given that 79.6% of the patients were female, a sex-stratified analysis was used to identify possible associations with *TSLP* polymorphisms. Table 3 shows significant differences in *TLSP* alleles between patients with GO and those without GO. Under a dominant model, we observed 1.52- and 1.7-fold increases in the risk of developing GO in female GD patients for particular genotypes at rs3806933 and rs2289278, respectively. For the C/T and C/C genotypes at rs2289276, we observed 6- and 4.93-fold increases in the risk of developing GO in female GD patients, respectively. Significant differences in *TSLP* genotype and allele frequencies were not observed in male GD patients (Table 3). Female GD patients harboring the G allele at rs2289278 were found to have a 1.56-fold increased risk for GO (Table 3).

**Table 3:** The genotype frequency of stratified by gender among Graves’ disease patients in Taiwan

**SNP ID**	**with GO**	**w/o GO**	***P *value**^a^	**OR (95% CI)**
	**N (%)**	**N (%)**		
**rs3806933**				
Female				
C/C	64 (42.7)	119 (53.1)		1
C/T	77 (51.3)	80 (35.7)		1.79 (1.16, 2.77)
T/T	9 (6)	25 (11.2)	0.007 (genotype)	0.67 (0.29, 1.52)
C/T + T/T	86 (57.3)	105 (46.9)	0.047 (dominant)	1.52 (1.00, 2.31)
C allele	205 (68.3)	318 (71.0)		1
T allele	95 (31.7)	130 (29.0)	0.44	1.13 (0.83, 1.56)
Male				
C/C	19 (37.3)	21 (46.7)		1
C/T	24 (47.1)	22 (48.9)		1.21 (0.52, 2.82)
T/T	8 (15.7)	2 (4.4)	0.18 (genotype)	4.42 (0.83, 23.47)
C/T + T/T	32 (62.7)	24 (53.3)	0.35 (dominant)	1.47 (0.65, 3.33)
C allele	62 (60.8)	64 (71.1)		1
T allele	40 (39.2)	26 (28.9)	0.13	1.59 (0.87, 2.91)
**rs2289276**				
Female				
T/T	2 (1.3)	15 (6.7)		1
C/T	60 (40.0)	75 (33.5)		6.00 (1.32, 27.27)
C/C	88 (58.7)	134 (59.8)	0.034 (genotype)	4.93 (1.10, 22.07)
C/T + C/C	148 (98.7)	209 (93.3)	0.015 (recessive)	5.31 (1.20, 23.57)
C allele	236 (78.7)	343 (76.6)		1
T allele	64 (21.3)	105 (23.4)	0.50	0.89 (0.62, 1.26)
Male				
T/T	3 (5.9)	4 (8.9)		1
C/T	17 (33.3)	15 (33.3)		1.51 (0.29, 7.87)
C/C	31 (60.8)	26 (57.8)	0.847 (genotype)	1.59 (0.33, 7.76)
C/T + C/C	48 (94.1)	41 (91.1)	0.572 (recessive)	1.56 (0.33, 7.38)
C allele	79 (77.5)	67 (74.4)		1
T allele	23 (22.5)	23 (25.6)	0.63	0.85 (0.44, 1.65)
**rs2289278**				
Female				
C/C	80 (53.3)	148 (66.1)		1
C/G	59 (39.3)	66 (29.5)		1.65 (1.06, 2.58)
G/G	11 (7.3)	10 (4.5)	0.042 (genotype)	2.04 (0.83, 5.00)
C/G + G/G	70 (46.7)	76 (33.9)	0.013 (dominant)	1.70 (1.12, 2.60)
C allele	219 (73.0)	362 (80.8)		1
G allele	81 (27.0)	86 (19.2)	0.012	1.56 (1.10, 2.20)
Male				
C/C	34 (66.7)	29 (64.4)		1
C/G	14 (27.5)	13 (28.9)		0.92 (0.37, 2.27)
G/G	3 (5.9)	3 (6.7)	0.971 (genotype)	0.85 (0.16, 4.55)
C/G + G/G	17 (33.3)	16 (35.6)	0.819 (dominant)	0.91 (0.39, 2.11)
C allele	82 (80.4)	71 (78.9)		1
G allele	20 (19.6)	19 (21.1)	0.80	0.91 (0.45, 1.84)

Abbreviations: CI, confidence interval; GO, Graves’ ophthalmopathy; SNP, single-nucleotide polymorphism. ^a^Dominant or recessive genotype frequencies were determined by chi-square test using 2 × 2 contingency tables. Genotype frequencies were determined by chi-square test using 2 × 3 contingency tables. Compared Graves’ diseases patients with and without GO. *P* values less than 0.016 were considered significant.

**Figure 1:**
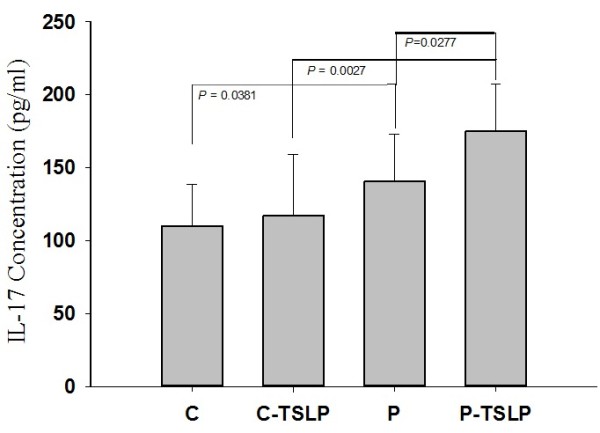
**TSLP increased Th17 differentiation in human PBMC isolated from 10 control subjects and 10 GD patients.** PBMC were activated by anti-CD3 and anti-CD28. Th17 differentiation was promoted by adding 10 ng/mL of TGF-β, 10 ng/mL of IL-23, 10 ng/mL of IL-6, 10 ng/mL of IL-1β, and 1 μg/mL of anti-IL4 and anti-IFNγ with (C-TSLP or P-TSLP), or without (C or P)125 ng/mL of TSLP. The concentrations of IL-17 in the culture supernatants were determined by enzyme-linked immunosorbent assay. C: control; P: patient. Values are mean ± standard deviation of 3 independent measurements. Student’s *t* tests have produced *p* values for the paired comparisons C versus P, C-TSLP versus P-TSLP, and P versus P-TSLP of 0.0381, 0.0027, and 0.0277, respectively.

Serum TSLP concentrations were determined in 272 control individuals, 184 GD patients with GO, and 248 GD patients without GO. The TSLP concentrations were significantly lower in the control subjects (*p* < 0.00001; Table 4). No significant differences were observed in TSLP expression levels between male and female patients or between patients with and without GO. Treating PBMC from control subjects and GD patients in vitro with higher TSLP concentrations increased IL-17 expression under conditions of Th17 differentiation. In the presence of IL-23, IL-6, IL-1β, TGF-β, TSLP, anti–IL-4, and anti–IFN-γ, the IL-17 expression levels increased with the TSLP concentration (Figure 1).

**Table 4:** Serum concentration of TSLP in control and Graves’ disease patients

***Samples***	***No.***	***TSLP concentration (pg/ml)***	***P***^***a***^	***P***^***b***^
**Control**	272	15.46 ± 35.25		
GD	432	109.93 ± 189.13	< 0.00001	
GD/GO	184	113.43 ± 221.44	< 0.00001	
GD/w/o GO	248	107.33 ± 161.47	< 0.00001	0.75
**Female**					
Control	166	17.90 ± 29.69			
GD	343	111.70 ± 189.30	< 0.00001		
GD/GO	138	114.55 ± 216.48	< 0.00001		
GD/w/o GO	205	109.77 ± 169.10	< 0.00001	0.83	
**Male**					
Control	106	11.64 ± 43.96			
GD	89	103.11 ± 189.38	< 0.00001		
GD/GO	46	110.06 ± 238.15	< 0.00001		
GD/w/o GO	43	95.68 ± 119.46	< 0.00001	0.72	

^a^*P* values were determined by unpaired *T* test to compare between control and GD, GD/GO, GD/w/o GO. ^b^*P* values were determined by unpaired *T* test to compare between GD/GO and GD/w/o GO. *P* values less than 0.05 were considered significant.

## Discussion

In this study, we demonstrated that *TSLP* polymorphisms are associated with GD and with GO in female GD patients, and that TSLP concentrations are increased in GD patients. We found an association between TSLP haplotypes and GD. Ht3 is a risk haplotype for GD, whereas Ht5 is a protective haplotype. No significant associations were observed between GD with GO and GD without GO before gender stratification. rs3806933, rs2289276, and rs2289278 showed significant associations with the presence or absence of GO in GD in female patients but not in male patients. This may be a result of the small number of male patients in our cohort. Increased expression TSLP levels induced CD4^+^ T cells to differentiate into Th17 cells, which are key players in GD development. IL-17 has a regulatory role in neutrophil recruitment and regulates the expression of inflammatory cytokines in epithelial cells, resulting in the induction of inflammation. The dysregulation of IL-17 production leads to chronic inflammation and tissue damage, both of which may lead to the development of GD.

The differentiation of Th17 cells is mediated by the combinatorial effects of TSLP, IL-23, TNF-α, TGF-β, IL-6, and IL-1β, which promote the expression of IL-17 [15]. In addition to *TSLP* polymorphisms, polymorphisms in the genes encoding IL-23 receptor [16], TGF-β (+869T/C and +915G/C) [17], IL-6 (+572G) [18], TNF-α (+1031C) [19], and 1 SNP in IL-1β (rs1143634) [20] were found to be associated with GD. Moreover, the serum expression levels of IL-6, IL-1β, and TNF-α were higher in GD patients compared with control subjects [21]. The increased expre-ssion of these proteins is believed to promote Th17 differentiation. These cytokines and chemokines are normally under strict control and do not produce disease symptoms under normal circumstances. However, some individuals are likely predisposed to cytokine or chemokine-mediated disease because of their genetic background. Individuals with GD may be genetically predisposed to produce greater quantities of TSLP, IL-6, IL-1β, and TNF-α and therefore, greater numbers of Th17 cells in the presence of appropriate stimuli. This genetic tendency may be regulated by polymorphisms in TSLP, IL-23, TGF-β, IL-6, IL-1β, and TNF-α.

We found that *TSLP* polymorphisms were only associated with GO in female patients. Approximately 78% of individuals with autoimmune disease are women. Women have increased antibody production (Th2 response) in response to infection or vaccination. In GD, the production of autoantibodies against thyrotropin receptor stimulates thyroid cells and contributes to the onset of the disease. Estrogen has been shown to increase Th2 response, whereas androgen increases Th1 response; this hormone-mediated difference may account for the higher number of female patients with GD. Given that only 96 male GD patients were included in this study, it is difficult to conclusively exclude a role for *TSLP* polymorphisms in the development of GD or GO in male patients.

Additional research is required to better understand the importance of TSLP in GD, particularly focusing on male GD patients. Ultimately, more clearly defining the roles of cytokine networks in the onset and pathogenesis of GD will aid the development of novel therapeutic regimens and treatment for patients who do not respond to existing therapies.

## Conclusions

The results obtained in this study suggest that the *TSLP* gene may be a relevant candidate gene for susceptibility to GD. *TSLP* genotypes may be used as genetic markers for the diagnosis and prognosis of GD. Furthermore, TLSP may be a target for treating GD.

## Competing interests

No authors have any financial/conflicting interests to disclose.

## Authors' contributions

KST and FJT performed the DNA extraction and prepared the draft of the manuscript. HJL and YHL selected polymorphisms and performed haplotype analysis. CCL performed the genotyping and ELISA experiments. LW and HJL analyzed the data and finalized the manuscript. All authors read and approved the final manuscript.

## Pre-publication history

The pre-publication history for this paper can be accessed here:

http://www.biomedcentral.com/1471-2350/13/116/prepub

## Supplementary Material

Additional file 1: Table S1 Demographic and clinical characteristics of Graves’ patients and control individuals. Click here for file
