# Supplementary material for: Thymic stromal lymphopoietin gene promoter polymorphisms and expression levels in Graves’ disease and Graves’ ophthalmopathy
Source: BMC Med Genet. 2012 Nov 30;13:116. doi: 10.1186/1471-2350-13-116 (PMC3582428; doi:10.1186/1471-2350-13-116)
Supplement: Additional file 1: Table S1 — Demographic and clinical characteristics of Graves’ patients and control individuals. [file 1471-2350-13-116-S1.doc]

**Table S1. Demographic and clinical characteristics of Graves’ patients and control individuals.**

| **Patients Characteristics** | **Healthy (Genotype)**  **(78)** | **GD**  **(470)** | **Healthy (TSLP)**  **(272)** | **GD (TSLP)**  **(432)** |
| --- | --- | --- | --- | --- |
| **Age at Diagnosis** |  |  |  |  |
| ≤ 40 | 50 (64.1%) | 337(71.7%) | 199 (73.2) | 300 (69.4%) |
| >40 | 28 (35.9%) | 133 (28.3%) | 73 (26.8) | 132 (30.6%) |
|  |  |  |  |  |
| **Gender** |  |  |  |  |
| Male | 19 (24.4%) | 96 (20.4%) | 106 (39.0) | 89 (20.6%) |
| Female | 59 (75.6%) | 374 (79.6%) | 166 (61.0) | 343 (79.4%) |
|  |  |  |  |  |
| **Treatment** |  |  |  |  |
| **Radioiodine** |  |  |  |  |
| No |  | 449 (95.5%) |  | 409 (94.7%) |
| Yes |  | 21 (4.5%) |  | 23 (5.3%) |
|  |  |  |  |  |
| **Thyroid gland surgery** |  |  |  |  |
| No |  | 423 (90.0%) |  | 390 (90.3%) |
| Yes |  | 47 (10.0%) |  | 42 (9.7%) |
|  |  |  |  |  |
| **Clinical features** |  |  |  |  |
| **Goiter** |  |  |  |  |
| Grade 1 |  | 31 (6.6%) |  | 26 (6.0%) |
| Grade 2 |  | 26 (5.5%) |  | 25 (5.8%) |
| Grade 3 |  | 52 (11.1%) |  | 42 (9.7%) |
| Grade 4 |  | 300 (63.8%) |  | 280 (64.8%) |
| Grade 5 |  | 61 (13.0%) |  | 59 (13.7%) |
|  |  |  |  |  |
| **Opthalmopathy** |  |  |  |  |
| No |  | 269 (57.2%) |  | 248 (57.4%) |
| Yes |  | 201 (42.8%) |  | 184 (42.6%) |
|  |  |  |  |  |
| **Nodular hyperplasia** |  |  |  |  |
| No |  | 422 (89.8%) |  | 390 (90.3%) |
| Yes |  | 48 (10.2%) |  | 42 (9.7%) |
|  |  |  |  |  |
| **Myxedema** |  |  |  |  |
| No |  | 464 (98.7%) |  | 429 (99.3%) |
| Yes |  | 6 (1.3%) |  | 3 (0.7%) |
|  |  |  |  |  |
| **Vitiligo** |  |  |  |  |
| No |  | 466 (99.1%) |  | 431 (99.8%) |
| Yes |  | 4 (0.9%) |  | 1 (0.2%) |
